# Oxy-Phosphate Cement

**Published:** 1883-11

**Authors:** 


### OXY-PHOSPHATE CEMENT.

Eccleston's Silicious oxy-phosphate cement is an excellent article. Many of the oxy-phosphate fillings are simply the calcined oxide of zinc, and a solution of glacial phosphoric acid. This is, according to the maker, a combination of oxide of zinc and silica, with borax calcined at a high heat, which vitrifies it into a mass that must be ground very fine, and then mixed with a proportion of calcined oxide of zinc, to form the powder. It works better than any oxy-phosphate that I have used, and sets exceedingly hard. What its durability may be can only, like other cements, be determined by time. C. F. W. B.

